# 12-Anilinomethyl-9α-hy­droxy-4,8-dimethyl-3,14-dioxatricyclo­[9.3.0.0^2,4^]tetra­dec-7-en-13-one

**DOI:** 10.1107/S1600536811015303

**Published:** 2011-05-11

**Authors:** Mohamed Moumou, Ahmed Benharref, Moha Berraho, Daniel Avignant, Abdelghani Oudahmane, Mohamed Akssira

**Affiliations:** a Laboratoire de Chimie Bioorganique et Analytique, URAC 22, BP 146, FSTM, Université Hassan II, Mohammedia-Casablanca 20810 Mohammedia, Morocco; b Laboratoire de Chimie des Substances Naturelles, URAC16, Faculté des Sciences Semlalia, BP 2390 Bd My Abdellah, 40000 Marrakech, Morocco; c Université Blaise Pascal, Laboratoire des Matériaux, Inorganiques, UMR CNRS 6002, 24 Avenue des Landais, 63177 Aubière, France

## Abstract

The title compound, C_21_H_27_NO_4_, was synthesized from 9α-hy­droxy­parthenolide, which was isolated from the chloro­form extract of the aerial parts of *Anvillea radiata*. The asymmetric unit contains two independent mol­ecules. In each, the ten-membered ring displays an approximative chair-chair conformation. Each of the five-membered rings adopts a flattened envelope conformation, the C(H)—C—C(H) atoms representing the flap lie out of the mean plane through the remaining four atoms by 0.443 (2) and 0.553 (2) Å. The dihedral angle between the least-squares planes through the ten- and five-membered rings in the two mol­ecules are similar [22.54 (17) and 23.39 (14)°]. In the crystal, mol­ecules are linked by O—H⋯O, O—H⋯N and N—H⋯O hydrogen bonds.

## Related literature

For the isolation and biological activity of 9α-hy­droxy­parthenolide, see: El Hassany *et al.* (2004 ▶). For the reactivity of this sesquiterpene, see: Castaneda-Acosta *et al.* (1997 ▶); Neukirch *et al.* (2003 ▶); Der-Ren *et al.* (2006 ▶); Neelakantan *et al.*(2009 ▶). For conformations of ten-membered rings, see: Watson & Zabel (1982 ▶). For conformational analysis, see: Cremer & Pople (1975 ▶).
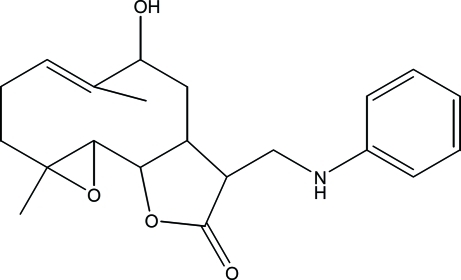


## Experimental

### 

#### Crystal data

C_21_H_27_NO_4_
*M* *_r_* = 357.44
Monoclinic, 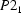

*a* = 11.1067 (8) Å
*b* = 11.9406 (9) Å
*c* = 14.6930 (11) Å
β = 106.315 (2)°
*V* = 1870.1 (2) Å^3^
*Z* = 4
Mo *K*α radiation
μ = 0.09 mm^−1^
*T* = 298 K
0.27 × 0.18 × 0.12 mm

#### Data collection

Bruker X8 APEXII CCD area-detector diffractometer
18091 measured reflections
4005 independent reflections
3651 reflections with *I* > 2σ(*I*)
*R*_int_ = 0.037

#### Refinement

*R*[*F* ^2^ > 2σ(*F* ^2^)] = 0.044
*wR*(*F* ^2^) = 0.131
*S* = 1.04
4005 reflections
474 parameters
1 restraint
H-atom parameters constrained
Δρ_max_ = 0.28 e Å^−3^
Δρ_min_ = −0.28 e Å^−3^

### 

Data collection: *APEX2* (Bruker, 2005 ▶); cell refinement: *SAINT* (Bruker, 2005 ▶); data reduction: *SAINT*; program(s) used to solve structure: *SHELXS97* (Sheldrick, 2008 ▶); program(s) used to refine structure: *SHELXL97* (Sheldrick, 2008 ▶); molecular graphics: *ORTEP-3* (Farrugia, 1997 ▶) and *PLATON* (Spek, 2009 ▶); software used to prepare material for publication: *WinGX* (Farrugia, 1999 ▶).

## Supplementary Material

Crystal structure: contains datablocks I, global. DOI: 10.1107/S1600536811015303/tk2738sup1.cif

Structure factors: contains datablocks I. DOI: 10.1107/S1600536811015303/tk2738Isup2.hkl

Supplementary material file. DOI: 10.1107/S1600536811015303/tk2738Isup3.cml

Additional supplementary materials:  crystallographic information; 3D view; checkCIF report

## Figures and Tables

**Table 1:** Hydrogen-bond geometry (Å, °)

*D*—H⋯*A*	*D*—H	H⋯*A*	*D*⋯*A*	*D*—H⋯*A*
N1—H1*A*⋯O6^i^	0.86	2.54	3.311 (3)	150
O1—H01⋯N2	0.82	2.41	3.221 (4)	169
O5—H05⋯O1	0.82	1.97	2.778 (3)	170

Symmetry code: (i) 
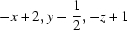
.

**Fig. 1.**
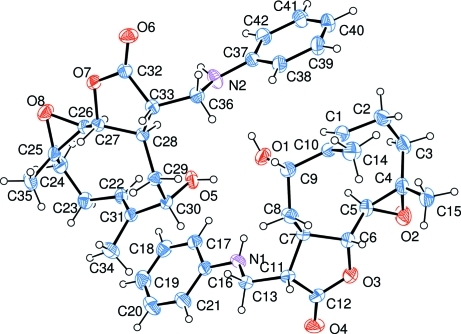
Molecular structure of the two independent molecules comprising the title compound with the atom-labelling scheme. Displacement ellipsoids are drawn at the 30% probability level. H atoms are represented as small spheres of arbitrary radii.

## supplementary crystallographic information

### Comment

Our work lies within the framework of the evaluation of medicinal plants and in particular, *Anvillea radiate*. The main constituent of the chloroform extract of aerial parts of this plant is 9α-hydroxypartenolide (El Hassany *et al.*, 2004). The reactivity of this sesquiterpene lactone and its derivatives has been the subject of several studies (Castaneda-Acosta *et al.*,1997; Neukirch *et al.*, 2003; Der-Ren *et al.*, 2006; Neelakantan *et al.*, 2009), in order to prepare high value added products for use in industrial pharmacology. In this context, we have treated 9α-hydroxyparthenolide with an equivalent amount of aniline in the presence of a catalytic amount of Lewis acid (ZnCl_2_) and isolated 9α -hydroxy-4,8-dimethyl-12-phenylaminomethyl-3,14-dioxa-tricyclo [9.3.0.0^2^,^4^] tetradec-7-in-13-one in a yield of 50%. The structure of this new product was confirmed by its single crystal X-ray structure.

The asymmetric unit contains two crystallographically independent molecules (Fig. 1). Each molecule is built up from two fused five- and ten-membered rings with the phenylaminomethyl group at positions 11 and 33 in the β configuration. The ten-membered ring displays an approximate chair-chair conformation. Whereas the five-membered rings shows an envelope conformation as indicated by Cremer & Pople (1975) puckering parameters Q = 0.275 (3) Å and φ = 72.1 (6) ° for the ring (C6,C7···O3), and Q = 0.350 (2) Å; φ = 74.7 (4)° for the other five membered ring (C27, C28···O7). The atoms C7 and C28 deviate from the respective mean plane through other four atoms in the ring by 0.443 (2) and 0.553 (2) Å respectively. In the first molecule (C1 to C21), the dihedral angle between the rings is 22.54 (17) °. The corresponding value in the second molecule (C22 to C42) is 23.39 (14) °. This is the typical conformation found for other sesquiterpenes lactones (Watson & Zabel, 1982). Intermolecular O—H···O(N) and N—H···O hydrogen bonds ensures the cohesion of the crystal structure, Table 1.

### Experimental

In a screw capped vial equipped with a magnetic stirrer, ZnCl_2_ (20 mg, 0.142 mmol) was added to aniline (270 mg, 2.91 mmol) and 9α-hydroxyparthenolide (753 mg, 2.85 mmol) in THF (5 ml). The resulting mixture was left under vigorous stirring at 40 °C for 10 days. The mixture was extracted with AcOEt (2 *x* 10 ml). The combined organic layers were dried over anhydrous Na_2_SO_4_ and concentrated under reduced pressure. Chromatography of the residue obtained on a column of silica gel eluting with hexane ethyl acetate (85/15) allowed the isolation of pure 9α -hydroxy-4,8-dimethyl-12-phenylaminomethyl-3,14-dioxa-tricyclo [9.3.0.0^2^,^4^]tetradec-7-en-13-one (508 mg, 1.42 mmol). The title compound was recrystallized from its ethyl acetate solution.

### Refinement

All H atoms were fixed geometrically and treated as riding with O—H = 0.82 Å, N—H = 0.86 Å and C—H = 0.93-0.98 Å, and with *U*_iso_(H) = 1.2*U*_eq_(O, N and C) or *U*_iso_(H) = 1.5*U*_eq_(methyl). In the absence of significant anomalous scattering, the absolute configuration could not be reliably determined and thus 3180 Friedel pairs were merged.

### Crystal data

**Table:**

C_21_H_27_NO_4_	*F*(000) = 768
*M**_r_* = 357.44	*D*_x_ = 1.270 Mg m^−^^3^
Monoclinic, *P*2_1_	Mo *K*α radiation, λ = 0.71073 Å
Hall symbol: P 2yb	Cell parameters from 18901 reflections
*a* = 11.1067 (8) Å	θ = 1.9–26.4°
*b* = 11.9406 (9) Å	µ = 0.09 mm^−^^1^
*c* = 14.6930 (11) Å	*T* = 298 K
β = 106.315 (2)°	Prism, colourless
*V* = 1870.1 (2) Å^3^	0.27 × 0.18 × 0.12 mm
*Z* = 4	

### Data collection

**Table:**

Bruker X8 APEXII CCD area-detector diffractometer	3651 reflections with *I* > 2σ(*I*)
Radiation source: fine-focus sealed tube	*R*_int_ = 0.037
graphite	θ_max_ = 26.4°, θ_min_ = 1.9°
φ and ω scans	*h* = −11→13
18091 measured reflections	*k* = −14→13
4005 independent reflections	*l* = −18→18

### Refinement

**Table:**

Refinement on *F*^2^	Secondary atom site location: difference Fourier map
Least-squares matrix: full	Hydrogen site location: inferred from neighbouring sites
*R*[*F*^2^ > 2σ(*F*^2^)] = 0.044	H-atom parameters constrained
*wR*(*F*^2^) = 0.131	*w* = 1/[σ^2^(*F*_o_^2^) + (0.1035*P*)^2^ + 0.0818*P*] where *P* = (*F*_o_^2^ + 2*F*_c_^2^)/3
*S* = 1.04	(Δ/σ)_max_ = 0.001
4005 reflections	Δρ_max_ = 0.28 e Å^−^^3^
474 parameters	Δρ_min_ = −0.28 e Å^−^^3^
1 restraint	Extinction correction: *SHELXL97* (Sheldrick, 2008), Fc^*^=kFc[1+0.001xFc^2^λ^3^/sin(2θ)]^-1/4^
Primary atom site location: structure-invariant direct methods	Extinction coefficient: 0.026 (4)

### Special details

**Table:**

Geometry. All s.u.'s (except the s.u. in the dihedral angle between two l.s. planes) are estimated using the full covariance matrix. The cell s.u.'s are taken into account individually in the estimation of s.u.'s in distances, angles and torsion angles; correlations between s.u.'s in cell parameters are only used when they are defined by crystal symmetry. An approximate (isotropic) treatment of cell s.u.'s is used for estimating s.u.'s involving l.s. planes.
Refinement. Refinement of *F*^2^ against ALL reflections. The weighted *R*-factor *wR* and goodness of fit *S* are based on *F*^2^, conventional *R*-factors *R* are based on *F*, with *F* set to zero for negative *F*^2^. The threshold expression of *F*^2^ > 2σ(*F*^2^) is used only for calculating *R*-factors(gt) *etc*. and is not relevant to the choice of reflections for refinement. *R*-factors based on *F*^2^ are statistically about twice as large as those based on *F*, and *R*- factors based on ALL data will be even larger.

### Fractional atomic coordinates and isotropic or equivalent isotropic displacement parameters (Å^2^)

**Table:**

	*x*	*y*	*z*	*U*_iso_*/*U*_eq_	
C1	0.7915 (3)	0.3012 (3)	0.74747 (18)	0.0488 (6)	
H1	0.8127	0.2537	0.7042	0.059*	
C2	0.7993 (3)	0.2517 (3)	0.8428 (2)	0.0614 (8)	
H2A	0.7910	0.3107	0.8860	0.074*	
H2B	0.8806	0.2167	0.8682	0.074*	
C3	0.6953 (3)	0.1641 (3)	0.8353 (2)	0.0660 (8)	
H3A	0.7121	0.0987	0.8016	0.079*	
H3B	0.6942	0.1409	0.8983	0.079*	
C4	0.5702 (3)	0.2127 (3)	0.78344 (19)	0.0540 (7)	
C5	0.5359 (3)	0.2050 (2)	0.67912 (19)	0.0476 (6)	
H5	0.5970	0.1650	0.6544	0.057*	
C6	0.4602 (2)	0.2914 (2)	0.61274 (18)	0.0464 (6)	
H6	0.4402	0.3543	0.6487	0.056*	
C7	0.5235 (2)	0.3342 (2)	0.53782 (15)	0.0390 (5)	
H7	0.5775	0.2749	0.5251	0.047*	
C8	0.5993 (3)	0.4438 (2)	0.55866 (19)	0.0506 (6)	
H8A	0.5947	0.4795	0.4985	0.061*	
H8B	0.5579	0.4931	0.5929	0.061*	
C9	0.7378 (3)	0.4360 (2)	0.61507 (18)	0.0503 (6)	
H9	0.7750	0.5102	0.6140	0.060*	
C10	0.7584 (2)	0.4040 (2)	0.71752 (17)	0.0440 (6)	
C11	0.4094 (2)	0.3478 (2)	0.45040 (17)	0.0423 (5)	
H11	0.3757	0.4229	0.4547	0.051*	
C12	0.3140 (2)	0.2666 (3)	0.4665 (2)	0.0520 (6)	
C13	0.4276 (2)	0.3389 (2)	0.35209 (17)	0.0456 (6)	
H13A	0.3464	0.3410	0.3052	0.055*	
H13B	0.4757	0.4028	0.3412	0.055*	
C14	0.7369 (3)	0.4992 (3)	0.7781 (2)	0.0616 (8)	
H14A	0.6497	0.5193	0.7593	0.092*	
H14B	0.7864	0.5625	0.7702	0.092*	
H14C	0.7610	0.4766	0.8434	0.092*	
C30	0.7524 (2)	0.4204 (2)	0.31645 (16)	0.0396 (5)	
H30	0.6674	0.4398	0.3184	0.047*	
C15	0.5203 (4)	0.3023 (4)	0.8337 (2)	0.0699 (9)	
H15A	0.4433	0.3309	0.7926	0.105*	
H15B	0.5805	0.3618	0.8509	0.105*	
H15C	0.5049	0.2718	0.8898	0.105*	
C16	0.4981 (2)	0.2039 (2)	0.25042 (18)	0.0445 (5)	
C17	0.5847 (3)	0.1218 (2)	0.2441 (2)	0.0501 (6)	
H17	0.6389	0.0930	0.2993	0.060*	
C18	0.5912 (3)	0.0826 (3)	0.1573 (2)	0.0586 (7)	
H18	0.6491	0.0273	0.1547	0.070*	
C31	0.7456 (2)	0.3887 (2)	0.21551 (15)	0.0392 (5)	
C19	0.5129 (4)	0.1244 (4)	0.0742 (2)	0.0729 (10)	
H19	0.5172	0.0979	0.0157	0.087*	
C20	0.4291 (4)	0.2054 (4)	0.0799 (2)	0.0813 (11)	
H20	0.3763	0.2344	0.0242	0.098*	
C21	0.4198 (3)	0.2465 (3)	0.1671 (2)	0.0617 (8)	
H21	0.3615	0.3018	0.1689	0.074*	
C22	0.8099 (2)	0.3026 (2)	0.19730 (17)	0.0423 (5)	
H22	0.8466	0.2560	0.2482	0.051*	
C23	0.8304 (3)	0.2713 (2)	0.10338 (19)	0.0509 (6)	
H23A	0.7758	0.3157	0.0532	0.061*	
H23B	0.8098	0.1930	0.0900	0.061*	
C24	0.9686 (3)	0.2921 (3)	0.10614 (19)	0.0571 (7)	
H24A	1.0216	0.2369	0.1468	0.068*	
H24B	0.9782	0.2839	0.0429	0.068*	
C25	1.0089 (2)	0.4075 (3)	0.14274 (16)	0.0492 (6)	
C26	1.0604 (2)	0.4175 (2)	0.24703 (16)	0.0421 (5)	
H26	1.0649	0.3465	0.2813	0.051*	
C27	1.0517 (2)	0.5191 (2)	0.30495 (15)	0.0381 (5)	
H27	1.0172	0.5826	0.2635	0.046*	
C28	0.9797 (2)	0.50289 (19)	0.37965 (14)	0.0344 (4)	
H28	0.9956	0.4266	0.4048	0.041*	
C29	0.8374 (2)	0.5233 (2)	0.35161 (17)	0.0402 (5)	
H29A	0.8175	0.5796	0.3021	0.048*	
H29B	0.8160	0.5543	0.4061	0.048*	
C33	1.0515 (2)	0.58488 (19)	0.45719 (15)	0.0365 (5)	
H33	1.0199	0.6602	0.4371	0.044*	
C32	1.1838 (2)	0.5786 (2)	0.44833 (16)	0.0396 (5)	
C35	0.9483 (3)	0.5033 (3)	0.08128 (19)	0.0646 (9)	
H35B	0.8588	0.4943	0.0637	0.097*	
H35C	0.9765	0.5050	0.0252	0.097*	
H35A	0.9706	0.5723	0.1155	0.097*	
C34	0.6593 (3)	0.4624 (3)	0.1425 (2)	0.0610 (8)	
H34C	0.5779	0.4635	0.1531	0.092*	
H34B	0.6525	0.4336	0.0803	0.092*	
H34A	0.6926	0.5371	0.1477	0.092*	
C36	1.0443 (2)	0.5693 (2)	0.55869 (16)	0.0415 (5)	
H36A	1.1009	0.6218	0.5999	0.050*	
H36B	0.9598	0.5858	0.5612	0.050*	
C37	1.0992 (2)	0.4316 (2)	0.68938 (16)	0.0404 (5)	
C38	1.0581 (3)	0.5010 (3)	0.75004 (17)	0.0485 (6)	
H38	1.0179	0.5680	0.7278	0.058*	
C39	1.0775 (3)	0.4699 (3)	0.8449 (2)	0.0605 (8)	
H39	1.0499	0.5168	0.8855	0.073*	
C40	1.1361 (3)	0.3719 (3)	0.8788 (2)	0.0667 (9)	
H40	1.1477	0.3516	0.9417	0.080*	
C41	1.1782 (3)	0.3030 (3)	0.8184 (2)	0.0638 (8)	
H41	1.2191	0.2365	0.8413	0.077*	
C42	1.1600 (3)	0.3320 (3)	0.7240 (2)	0.0520 (6)	
H42	1.1884	0.2849	0.6840	0.062*	
N1	0.4918 (2)	0.2371 (2)	0.34005 (15)	0.0489 (5)	
H1A	0.5266	0.1966	0.3887	0.059*	
N2	1.0773 (3)	0.45606 (19)	0.59289 (14)	0.0527 (6)	
H2	1.0834	0.4041	0.5539	0.063*	
O1	0.7980 (2)	0.3611 (2)	0.56602 (15)	0.0659 (7)	
H01	0.8724	0.3776	0.5775	0.099*	
O2	0.4732 (3)	0.1356 (2)	0.73250 (17)	0.0710 (7)	
O3	0.34515 (19)	0.2358 (2)	0.55866 (15)	0.0637 (6)	
O4	0.2194 (2)	0.2324 (3)	0.41145 (19)	0.0765 (8)	
O5	0.7933 (2)	0.32770 (17)	0.37792 (12)	0.0524 (5)	
H05	0.7965	0.3458	0.4324	0.079*	
O6	1.28143 (17)	0.59853 (19)	0.50680 (14)	0.0552 (5)	
O7	1.18128 (16)	0.54301 (16)	0.36102 (11)	0.0430 (4)	
O8	1.14237 (18)	0.4236 (2)	0.18592 (14)	0.0618 (6)	

### Atomic displacement parameters (Å^2^)

**Table:**

	*U*^11^	*U*^22^	*U*^33^	*U*^12^	*U*^13^	*U*^23^
C1	0.0482 (13)	0.0575 (15)	0.0426 (12)	0.0078 (12)	0.0162 (10)	−0.0083 (11)
C2	0.0603 (17)	0.073 (2)	0.0473 (14)	0.0220 (16)	0.0093 (12)	0.0034 (14)
C3	0.082 (2)	0.0701 (19)	0.0481 (14)	0.0188 (18)	0.0226 (14)	0.0164 (14)
C4	0.0630 (16)	0.0602 (16)	0.0436 (13)	−0.0008 (14)	0.0229 (12)	0.0092 (12)
C5	0.0555 (15)	0.0461 (13)	0.0459 (12)	−0.0014 (12)	0.0222 (11)	0.0029 (11)
C6	0.0454 (13)	0.0529 (14)	0.0432 (12)	−0.0018 (12)	0.0163 (10)	0.0016 (11)
C7	0.0346 (11)	0.0442 (12)	0.0369 (10)	−0.0007 (10)	0.0081 (8)	0.0010 (9)
C8	0.0507 (14)	0.0475 (14)	0.0472 (12)	−0.0077 (12)	0.0035 (11)	0.0057 (11)
C9	0.0454 (14)	0.0571 (15)	0.0467 (13)	−0.0132 (12)	0.0101 (11)	−0.0005 (12)
C10	0.0354 (11)	0.0552 (14)	0.0414 (12)	−0.0021 (11)	0.0107 (9)	−0.0111 (11)
C11	0.0328 (11)	0.0484 (13)	0.0443 (12)	0.0039 (10)	0.0088 (9)	0.0043 (10)
C12	0.0338 (12)	0.0651 (17)	0.0575 (15)	−0.0002 (12)	0.0135 (11)	0.0071 (13)
C13	0.0384 (11)	0.0562 (15)	0.0404 (12)	0.0022 (11)	0.0079 (9)	0.0050 (11)
C14	0.0629 (17)	0.0616 (17)	0.0625 (16)	0.0081 (15)	0.0211 (14)	−0.0163 (14)
C30	0.0348 (11)	0.0490 (13)	0.0375 (11)	−0.0047 (10)	0.0144 (8)	0.0006 (10)
C15	0.072 (2)	0.096 (3)	0.0502 (14)	0.0136 (19)	0.0310 (14)	0.0030 (17)
C16	0.0385 (12)	0.0522 (14)	0.0425 (12)	−0.0119 (11)	0.0110 (9)	0.0012 (10)
C17	0.0448 (13)	0.0544 (15)	0.0503 (13)	−0.0073 (12)	0.0120 (11)	−0.0018 (12)
C18	0.0538 (15)	0.0599 (17)	0.0671 (17)	−0.0124 (14)	0.0251 (13)	−0.0137 (14)
C31	0.0365 (11)	0.0467 (12)	0.0340 (10)	−0.0103 (10)	0.0095 (8)	−0.0004 (9)
C19	0.081 (2)	0.091 (3)	0.0499 (15)	−0.012 (2)	0.0235 (15)	−0.0146 (17)
C20	0.090 (2)	0.107 (3)	0.0412 (15)	0.006 (2)	0.0083 (15)	0.0066 (18)
C21	0.0651 (18)	0.0718 (19)	0.0450 (14)	0.0087 (16)	0.0100 (12)	0.0063 (13)
C22	0.0474 (13)	0.0432 (12)	0.0373 (11)	−0.0073 (11)	0.0132 (9)	0.0005 (9)
C23	0.0587 (15)	0.0519 (14)	0.0432 (12)	−0.0102 (13)	0.0160 (11)	−0.0105 (11)
C24	0.0625 (16)	0.0734 (19)	0.0391 (12)	−0.0036 (15)	0.0204 (11)	−0.0149 (13)
C25	0.0463 (13)	0.0719 (18)	0.0335 (11)	−0.0125 (13)	0.0179 (10)	−0.0062 (11)
C26	0.0382 (11)	0.0553 (14)	0.0336 (10)	−0.0017 (11)	0.0113 (9)	−0.0008 (10)
C27	0.0339 (11)	0.0470 (12)	0.0319 (10)	−0.0038 (10)	0.0065 (8)	0.0030 (9)
C28	0.0363 (10)	0.0343 (10)	0.0320 (9)	−0.0009 (9)	0.0088 (8)	0.0007 (8)
C29	0.0382 (11)	0.0421 (12)	0.0406 (11)	0.0012 (10)	0.0115 (9)	−0.0032 (9)
C33	0.0394 (11)	0.0337 (10)	0.0360 (10)	−0.0019 (9)	0.0097 (8)	−0.0016 (9)
C32	0.0402 (12)	0.0379 (11)	0.0400 (11)	−0.0043 (10)	0.0100 (9)	−0.0019 (9)
C35	0.077 (2)	0.081 (2)	0.0340 (12)	−0.0202 (18)	0.0122 (12)	0.0104 (13)
C34	0.0603 (17)	0.0706 (19)	0.0451 (13)	0.0094 (16)	0.0032 (12)	0.0035 (13)
C36	0.0486 (12)	0.0393 (12)	0.0369 (11)	−0.0018 (10)	0.0122 (9)	−0.0047 (9)
C37	0.0401 (12)	0.0414 (12)	0.0382 (11)	−0.0087 (10)	0.0085 (9)	0.0007 (9)
C38	0.0510 (14)	0.0539 (15)	0.0423 (12)	−0.0043 (12)	0.0160 (10)	−0.0003 (11)
C39	0.0647 (18)	0.078 (2)	0.0449 (13)	−0.0093 (16)	0.0255 (13)	−0.0008 (14)
C40	0.0673 (19)	0.088 (2)	0.0457 (14)	−0.0106 (18)	0.0168 (13)	0.0184 (15)
C41	0.0653 (18)	0.0669 (18)	0.0585 (16)	0.0050 (16)	0.0165 (14)	0.0261 (15)
C42	0.0574 (16)	0.0480 (14)	0.0508 (14)	0.0011 (12)	0.0155 (11)	0.0030 (12)
N1	0.0478 (11)	0.0582 (13)	0.0358 (9)	0.0090 (10)	0.0038 (8)	0.0039 (9)
N2	0.0830 (17)	0.0409 (11)	0.0326 (9)	0.0061 (11)	0.0136 (10)	−0.0028 (9)
O1	0.0464 (10)	0.1072 (19)	0.0495 (10)	−0.0175 (12)	0.0223 (9)	−0.0218 (12)
O2	0.0815 (16)	0.0694 (14)	0.0646 (13)	−0.0191 (12)	0.0244 (11)	0.0158 (11)
O3	0.0457 (10)	0.0896 (17)	0.0575 (11)	−0.0153 (11)	0.0173 (9)	0.0120 (11)
O4	0.0414 (11)	0.0991 (19)	0.0785 (15)	−0.0171 (12)	−0.0001 (10)	0.0144 (14)
O5	0.0671 (12)	0.0554 (11)	0.0368 (8)	−0.0122 (9)	0.0182 (8)	0.0066 (8)
O6	0.0402 (9)	0.0663 (12)	0.0538 (10)	−0.0085 (9)	0.0047 (8)	−0.0156 (9)
O7	0.0371 (8)	0.0533 (10)	0.0392 (8)	−0.0073 (8)	0.0120 (6)	−0.0037 (7)
O8	0.0455 (10)	0.0931 (17)	0.0533 (10)	−0.0095 (11)	0.0244 (8)	−0.0165 (11)

### Geometric parameters (Å, °)

**Table:**

C1—C10	1.320 (4)	C19—H19	0.9300
C1—C2	1.500 (4)	C20—C21	1.403 (5)
C1—H1	0.9300	C20—H20	0.9300
C2—C3	1.539 (5)	C21—H21	0.9300
C2—H2A	0.9700	C22—C23	1.507 (3)
C2—H2B	0.9700	C22—H22	0.9300
C3—C4	1.501 (5)	C23—C24	1.544 (4)
C3—H3A	0.9700	C23—H23A	0.9700
C3—H3B	0.9700	C23—H23B	0.9700
C4—O2	1.454 (4)	C24—C25	1.501 (4)
C4—C5	1.475 (4)	C24—H24A	0.9700
C4—C15	1.492 (5)	C24—H24B	0.9700
C5—O2	1.446 (3)	C25—O8	1.452 (3)
C5—C6	1.504 (4)	C25—C26	1.482 (3)
C5—H5	0.9800	C25—C35	1.495 (5)
C6—O3	1.461 (3)	C26—O8	1.449 (3)
C6—C7	1.550 (3)	C26—C27	1.500 (4)
C6—H6	0.9800	C26—H26	0.9800
C7—C11	1.539 (3)	C27—O7	1.471 (3)
C7—C8	1.539 (4)	C27—C28	1.541 (3)
C7—H7	0.9800	C27—H27	0.9800
C8—C9	1.532 (4)	C28—C29	1.537 (3)
C8—H8A	0.9700	C28—C33	1.543 (3)
C8—H8B	0.9700	C28—H28	0.9800
C9—O1	1.427 (4)	C29—H29A	0.9700
C9—C10	1.507 (4)	C29—H29B	0.9700
C9—H9	0.9800	C33—C32	1.513 (3)
C10—C14	1.504 (4)	C33—C36	1.527 (3)
C11—C12	1.503 (4)	C33—H33	0.9800
C11—C13	1.518 (3)	C32—O6	1.203 (3)
C11—H11	0.9800	C32—O7	1.344 (3)
C12—O4	1.203 (4)	C35—H35B	0.9600
C12—O3	1.351 (4)	C35—H35C	0.9600
C13—N1	1.444 (4)	C35—H35A	0.9600
C13—H13A	0.9700	C34—H34C	0.9600
C13—H13B	0.9700	C34—H34B	0.9600
C14—H14A	0.9600	C34—H34A	0.9600
C14—H14B	0.9600	C36—N2	1.453 (3)
C14—H14C	0.9600	C36—H36A	0.9700
C30—O5	1.420 (3)	C36—H36B	0.9700
C30—C31	1.512 (3)	C37—C38	1.385 (4)
C30—C29	1.548 (3)	C37—C42	1.391 (4)
C30—H30	0.9800	C37—N2	1.400 (3)
C15—H15A	0.9600	C38—C39	1.400 (4)
C15—H15B	0.9600	C38—H38	0.9300
C15—H15C	0.9600	C39—C40	1.363 (5)
C16—C21	1.384 (4)	C39—H39	0.9300
C16—C17	1.395 (4)	C40—C41	1.384 (5)
C16—N1	1.396 (3)	C40—H40	0.9300
C17—C18	1.380 (4)	C41—C42	1.388 (4)
C17—H17	0.9300	C41—H41	0.9300
C18—C19	1.377 (5)	C42—H42	0.9300
C18—H18	0.9300	N1—H1A	0.8600
C31—C22	1.321 (4)	N2—H2	0.8600
C31—C34	1.505 (4)	O1—H01	0.8200
C19—C20	1.361 (6)	O5—H05	0.8200
			
C10—C1—C2	127.9 (3)	C16—C21—C20	119.3 (3)
C10—C1—H1	116.0	C16—C21—H21	120.3
C2—C1—H1	116.0	C20—C21—H21	120.3
C1—C2—C3	110.8 (3)	C31—C22—C23	127.4 (2)
C1—C2—H2A	109.5	C31—C22—H22	116.3
C3—C2—H2A	109.5	C23—C22—H22	116.3
C1—C2—H2B	109.5	C22—C23—C24	109.9 (2)
C3—C2—H2B	109.5	C22—C23—H23A	109.7
H2A—C2—H2B	108.1	C24—C23—H23A	109.7
C4—C3—C2	110.1 (3)	C22—C23—H23B	109.7
C4—C3—H3A	109.6	C24—C23—H23B	109.7
C2—C3—H3A	109.6	H23A—C23—H23B	108.2
C4—C3—H3B	109.6	C25—C24—C23	110.6 (3)
C2—C3—H3B	109.6	C25—C24—H24A	109.5
H3A—C3—H3B	108.2	C23—C24—H24A	109.5
O2—C4—C5	59.19 (18)	C25—C24—H24B	109.5
O2—C4—C15	112.6 (3)	C23—C24—H24B	109.5
C5—C4—C15	122.4 (3)	H24A—C24—H24B	108.1
O2—C4—C3	117.6 (3)	O8—C25—C26	59.19 (15)
C5—C4—C3	115.8 (3)	O8—C25—C35	113.2 (3)
C15—C4—C3	116.5 (3)	C26—C25—C35	122.7 (3)
O2—C5—C4	59.69 (19)	O8—C25—C24	116.3 (3)
O2—C5—C6	118.5 (2)	C26—C25—C24	115.7 (2)
C4—C5—C6	124.4 (3)	C35—C25—C24	116.6 (2)
O2—C5—H5	114.4	O8—C26—C25	59.37 (15)
C4—C5—H5	114.4	O8—C26—C27	117.5 (2)
C6—C5—H5	114.4	C25—C26—C27	125.7 (2)
O3—C6—C5	106.6 (2)	O8—C26—H26	114.2
O3—C6—C7	105.40 (19)	C25—C26—H26	114.2
C5—C6—C7	113.9 (2)	C27—C26—H26	114.2
O3—C6—H6	110.3	O7—C27—C26	105.43 (19)
C5—C6—H6	110.3	O7—C27—C28	104.31 (16)
C7—C6—H6	110.3	C26—C27—C28	115.5 (2)
C11—C7—C8	110.6 (2)	O7—C27—H27	110.4
C11—C7—C6	101.32 (19)	C26—C27—H27	110.4
C8—C7—C6	118.1 (2)	C28—C27—H27	110.4
C11—C7—H7	108.8	C29—C28—C27	119.32 (18)
C8—C7—H7	108.8	C29—C28—C33	112.10 (19)
C6—C7—H7	108.8	C27—C28—C33	100.51 (17)
C9—C8—C7	117.8 (2)	C29—C28—H28	108.1
C9—C8—H8A	107.9	C27—C28—H28	108.1
C7—C8—H8A	107.9	C33—C28—H28	108.1
C9—C8—H8B	107.9	C28—C29—C30	116.7 (2)
C7—C8—H8B	107.9	C28—C29—H29A	108.1
H8A—C8—H8B	107.2	C30—C29—H29A	108.1
O1—C9—C10	112.6 (2)	C28—C29—H29B	108.1
O1—C9—C8	107.9 (2)	C30—C29—H29B	108.1
C10—C9—C8	113.7 (2)	H29A—C29—H29B	107.3
O1—C9—H9	107.4	C32—C33—C36	113.35 (19)
C10—C9—H9	107.4	C32—C33—C28	102.57 (18)
C8—C9—H9	107.4	C36—C33—C28	119.1 (2)
C1—C10—C14	125.5 (3)	C32—C33—H33	107.1
C1—C10—C9	121.2 (2)	C36—C33—H33	107.1
C14—C10—C9	113.3 (3)	C28—C33—H33	107.1
C12—C11—C13	113.1 (2)	O6—C32—O7	121.1 (2)
C12—C11—C7	104.6 (2)	O6—C32—C33	129.1 (2)
C13—C11—C7	119.2 (2)	O7—C32—C33	109.72 (18)
C12—C11—H11	106.4	C25—C35—H35B	109.5
C13—C11—H11	106.4	C25—C35—H35C	109.5
C7—C11—H11	106.4	H35B—C35—H35C	109.5
O4—C12—O3	120.7 (3)	C25—C35—H35A	109.5
O4—C12—C11	129.4 (3)	H35B—C35—H35A	109.5
O3—C12—C11	109.9 (2)	H35C—C35—H35A	109.5
N1—C13—C11	112.2 (2)	C31—C34—H34C	109.5
N1—C13—H13A	109.2	C31—C34—H34B	109.5
C11—C13—H13A	109.2	H34C—C34—H34B	109.5
N1—C13—H13B	109.2	C31—C34—H34A	109.5
C11—C13—H13B	109.2	H34C—C34—H34A	109.5
H13A—C13—H13B	107.9	H34B—C34—H34A	109.5
C10—C14—H14A	109.5	N2—C36—C33	111.98 (19)
C10—C14—H14B	109.5	N2—C36—H36A	109.2
H14A—C14—H14B	109.5	C33—C36—H36A	109.2
C10—C14—H14C	109.5	N2—C36—H36B	109.2
H14A—C14—H14C	109.5	C33—C36—H36B	109.2
H14B—C14—H14C	109.5	H36A—C36—H36B	107.9
O5—C30—C31	110.4 (2)	C38—C37—C42	119.3 (2)
O5—C30—C29	110.49 (18)	C38—C37—N2	122.1 (2)
C31—C30—C29	112.54 (19)	C42—C37—N2	118.5 (2)
O5—C30—H30	107.8	C37—C38—C39	119.8 (3)
C31—C30—H30	107.8	C37—C38—H38	120.1
C29—C30—H30	107.8	C39—C38—H38	120.1
C4—C15—H15A	109.5	C40—C39—C38	121.0 (3)
C4—C15—H15B	109.5	C40—C39—H39	119.5
H15A—C15—H15B	109.5	C38—C39—H39	119.5
C4—C15—H15C	109.5	C39—C40—C41	119.3 (3)
H15A—C15—H15C	109.5	C39—C40—H40	120.4
H15B—C15—H15C	109.5	C41—C40—H40	120.4
C21—C16—C17	118.2 (3)	C40—C41—C42	120.8 (3)
C21—C16—N1	123.0 (3)	C40—C41—H41	119.6
C17—C16—N1	118.8 (2)	C42—C41—H41	119.6
C18—C17—C16	121.1 (3)	C41—C42—C37	119.9 (3)
C18—C17—H17	119.5	C41—C42—H42	120.1
C16—C17—H17	119.5	C37—C42—H42	120.1
C19—C18—C17	120.8 (3)	C16—N1—C13	120.8 (2)
C19—C18—H18	119.6	C16—N1—H1A	119.6
C17—C18—H18	119.6	C13—N1—H1A	119.6
C22—C31—C34	125.7 (2)	C37—N2—C36	120.0 (2)
C22—C31—C30	120.8 (2)	C37—N2—H2	120.0
C34—C31—C30	113.5 (2)	C36—N2—H2	120.0
C20—C19—C18	118.4 (3)	C9—O1—H01	109.5
C20—C19—H19	120.8	C5—O2—C4	61.12 (17)
C18—C19—H19	120.8	C12—O3—C6	111.1 (2)
C19—C20—C21	122.1 (3)	C30—O5—H05	109.5
C19—C20—H20	118.9	C32—O7—C27	110.52 (18)
C21—C20—H20	118.9	C26—O8—C25	61.44 (15)

### Hydrogen-bond geometry (Å, °)

**Table:**

*D*—H···*A*	*D*—H	H···*A*	*D*···*A*	*D*—H···*A*
N1—H1A···O6^i^	0.86	2.54	3.311 (3)	150
O1—H01···N2	0.82	2.41	3.221 (4)	169
O5—H05···O1	0.82	1.97	2.778 (3)	170

Symmetry codes: (i) −*x*+2, *y*−1/2, −*z*+1.

## References

[bb1] Bruker, (2005). *APEX2* and *SAINT* Bruker AXS Inc., Madison, Wisconsin, USA.

[bb2] Castaneda-Acosta, J., Pentes, H. G., Fronczek, F. R. & Fischer, N. H. (1997). *J. Chem. Crystallogr.* **27**, 635–639.

[bb3] Cremer, D. & Pople, J. A. (1975). *J. Am. Chem. Soc.* **97**, 1354–1358.

[bb4] Der-Ren, H., Yu-Shan, W., Chun-Wei, C., Tzu-Wen, L., Wei-Cheng, C., Uan-Kang, T., John, T. A. H. & Hsing-Pang, H. (2006). *Bioorg. Med. Chem. Lett.* **14**, 83–91.

[bb5] El Hassany, B., El Hanbali, F., Akssira, M., Mellouki, F., Haidou, A. & Barero, A. F. (2004). *Fitoterapia*, **75**, 573–576. 10.1016/j.fitote.2004.06.003 15351111

[bb6] Farrugia, L. J. (1997). *J. Appl. Cryst.* **30**, 565.

[bb7] Farrugia, L. J. (1999). *J. Appl. Cryst.* **32**, 837–838.

[bb8] Neelakantan, S., Nasim, Sh., Guzman, M. L., Jordan, C. T. & Crooks, P. A. (2009). *Bioorg. Med. Chem. Lett.* **19**, 4346–4349. 10.1016/j.bmcl.2009.05.092 19505822

[bb9] Neukirch, H., Kaneider, N. C., Wiedermann, C. J., Guerriero, A. & Ambrosio, M. (2003). *Bioorg. Med. Chem.* **11**, 1503–1510. 10.1016/s0968-0896(02)00553-9 12628675

[bb10] Sheldrick, G. M. (2008). *Acta Cryst.* A**64**, 112–122. 10.1107/S0108767307043930 18156677

[bb11] Spek, A. L. (2009). *Acta Cryst.* D**65**, 148–155. 10.1107/S090744490804362X PMC2631630 19171970

[bb12] Watson, W. H. & Zabel, V. (1982). *Acta Cryst.* B**38**, 834–838.

